# The Present Treatment of Adenoid Vegetations

**Published:** 1890-09

**Authors:** B. M. Behrens

**Affiliations:** Chicago, Ill.


### THE PRESENT TREATMENT OF ADENOID VEGETATIONS.*

BY B. M. BEHRENS, M. D., CHICAGO, ILL.

I have for a long time thought of presenting this paper to the medical fraternity, but so much has been written upon this topic of late, and I dare say so much conquered, that there seemed to me to be no haste about it, as I might find my views better presented by somebody else. The heading of my paper indicates its contents and is meant principally to be a critique upon the existing treatment more than a suggestion of any new one, what we are really not in need of, if we only select the most radical and most feasible of those proceedings so often mentioned by others. When I here propose to show the inadequate progress of the therapeutics in proportion to the wide view of the pathological and clinical aspects, I trust that you will, before I have finished, agree with me upon the weak points of the situation. You know that rhinology has of late conquered more points than any other branch of medicine, I dare say, not only in regard to recognizing near and dire consequences of a diseased or abnormal condition of the nostrils and the post-nasal space, but also in regard to more remote symptoms, that were, not long ago, never attributed to such obscure regions as those above mentioned. Here is opened a field where many and honest workers are occupied in enlarging our knowledge and bringing forth every once in a while new facts, concerning which physicians of earlier date knew very little or nothing. I shall only call to your mind frequent contributions in the medical journals upon this topic, and the many very able discussions, we have listened to in this Society. It would lead me too far to recapitulate it, and therefore I shall refer those, who want a synopsis of it, to read a very able article on this topic, which will be found in the first

*Read before Chicago Medical Society, June 2, 1890.

March copy of the *Journal of the American Medical Association*. Dr. Delavan calls our attention here to all those anomalies, caused by adenoid vegetations, from assymetry of the face, pinched nostrils, open mouth, projecting teeth, arched palate, stupid expression, anæmic surface, drooping eyelids to the stenosis of one or both nostrils, with constant catarrh and defective speaking, discharge from the ears, loss of hearing, mental dulness, deformity of the chest and so forth. If the adenoid vegetations in the post-nasal space are the root to all these evils, what I don't doubt, then it is time, fellow physicians, to give the child a chance. He says it is one of the most annoying and pernicious conditions which we are called upon to treat. I fully agree with him, and I would add, that it would be almost criminal carelessness on the part of that physician, who would say, that these things will outgrow themselves. Certainly they do in many cases, but too late after they have done irreparable harm. For the sake of illustrating it, I should like to know how many small ones have lost their hearing forever by this popular trash. In my practice I have seen too many deplorable consequences of this foolish neglect; but luckily-wise the light is spreading, and the children will reap the blessing. How? That is the question we have to meet, not by paraphernalia by the dozen, but by single means that are at hand to us all. To make the physicians sick of a take on children's ills and miseries, and, at the same time, surround the possible help for it with a Chinese wall, which only the most able specialists can climb, is but to evade the question. We must remember that we here speak about common ills, where the common practitioner ought to be enabled to lend a helping hand, or rather finger. In my practice of fifteen years I have employed only this—the index—in children, and the ring-knife invented by Dr. Meyer, of Copenhagen, in adults, and I defy any specialist with his rhinoscopic mirror, mouth-gag, palate-retractor, different instruments, chloroform and assistants, to show me better results than I have obtained. To hunt (?) for instruments and means that only lead to a further complication of the already too complicated treatment serves only to make it more unsavory than it already is. And here is the point where all the able writers fail to harmonize theory with practice. Instead of looking around for a simple proceeding, that don't need to have even an appearance of operating, an effort is made to make it look like a big undertaking. What for? And the result is no better. Is there any objection whatever to the simple proceeding ? Not at all. The first thing we consult is the result. As said it is as good after employing the finger-nail as after employing the most handy instruments. Is there more pain connected with using the finger-nail ? Not at all. If we want to, we can apply cocaine to all the parts that we are likely to touch with the finger, to lessen the pain in a considerable degree. Is it likely to be more repulsive to the child than a display of ugly instruments ? Certainly not. Is it likely that we can do more harm with the finger-nail than with instruments. Most certainly not. I remember only a few instances of objections to employing the finger ; but on the other side, how many would be willing to submit a second time to the cruel instruments and the other devices above referred to. The fact is, we cannot and ought not to remove all the mass we may find in the pharyngeal vault in one sitting, not only because of the nervous shock that might accompany such a procedure, but also and more because an operation to such an extent would give us so large a wound surface for the after treatment that we would lose control of it in a locality where we can hardly apply any disinfectants at all. Preferable is it on this account also to do the required work in more sittings, as the scraping with the finger-nail need only occupy half a minute to one minute, or even a shorter time, according to the texture of the vegetations and the dexterity of the operator. As these are always soft in children it is a very easy matter to scrape away a good deal at once. Usually the children declare right off that they can breath easily through the corresponding nostril. The often very profuse bleeding is easily checked with a lukewarm nose-douche of salt water. The operation on the other side can be repeated eight to ten days after and the nose-douche again applied. To be sure that all, or almost all, is removed, I go over this field once more after eight to ten days, so that the whole undertaking takes three to four weeks, in which the child scarcely notices any trouble whatever, save for some accumulation of mucus or pus. My small patients have even always attended school in that time. In regard to the absolute necessity of removing all, so that not the smallest particles of these vegetations are left, then I cannot agree upon this claim. This is also a part of the Chinese wall, with which the undertaking is surrounded to deprive most of the physicians of the little courage they might have to do something. To free the posterior openings of the nostrils and the orifices for tube Eustachii is our aim, no matter whether there is left a small knot of adenoid tissue or not. Just as well might be claimed the absolute necessity of removing small hypertrophic follicles, that we always find dispersed here and there on the posterior pharyngeal wall. My proceeding is soon described. I do not waste any time with rhinoscopy or the like. The diagnosis can be made without—and I wonder what the rhinoscopist can see—but simply ask the patient to open the mouth and put my index finger along the palate, until I feel with the tip the free edge of the soft palate, and in bending the finger lower the hand, after which I softly glide the finger upwards to the soft mass and scrape at once. With my other free hand I press the patient's head in to me and downwards to prevent the patient raising from the chair. All the assistance I ask for is one to hold the patient's hands, so he can't interfere. Of course, it doesn't go without some little fighting, but as remarked above, the patients don't object to a repetition another time. It can be done so gently as to find no resistance even in small children. The youngest ones I have operated upon were three years of age, and where a display of ugly instruments would take the wits out of them. It is evident, that it will not do for a giant with a fist for base-ball to put his finger into a little child's throat and with rough force fight his way behind the soft palate. It must be left to a small hand and neat finger to do this kind of work.

When the fibrous tissue is more predominant and consequently the vegetations denser (harder), or in adults, I mostly employ the ring-knife. As you will see, gentlemen, it is only a curette with a straight handle, that is introduced in the nostril corresponding to that side of the post-nasal space, where we want to operate. In all text-books, and partly papers, there is evidently a misconception of the construction of this instrument. I have seen it somewhere described as a curved curette, or as a lithotome-like contrivance, but as you see here, it is neither. It is not introduced through the mouth, but through the nostril, while the index finger of the operator's other hand is introduced behind the soft palate to lead the ring in its scraping work. Whatever I employ, the finger-nail or the ring-knife, I always apply a two to four per cent. solution of cocaine to the soft palate, and for the post-nasal space a cotton pellet, on a bent cotton-holder, saturated with the solution ; for the nostril a spray not only to alleviate the pain, but also to make the nostril wider and easier passable to the ring-knife. After the operation I use a nose douche of lukewarm salt water, that does away with loose tumor rests and blood, and is applied to the most obstructed nostril, to prevent any accident to the ears. As a last act I apply some iodoform on the post-nasal space for the sake of a good conscience, and I have never had any cause whatever from any point of view to prefer any other way of operating on this pernicious condition, we call adenoid vegetations.

It is a matter of course, that this proceeding also requires some dexterity on the part of the operator, not only to do the work well, but also to do it in such an effective, and at the same time, lenient way as to win the confidence of the patients ; that is indespensable considering the necessity of doing the operation several times.

All writers lay much stress upon the necessity of removing all of the adenoid tissue in the pharyngeal vault. To me they look as conscientious physicians, who wish to do the thing very efficiently, and if they cannot, they prefer not to do anything at all. There are many tonsillotomists, who claim that an entire removal of hypertrophied tonsils is indispensable to be effective, and apply the same reasoning to the adenoid tumors. This is a great mistake. These tumors are as harmless as small tonsils. It is first, when they are present in such multitude as to obstruct the posterior nares, that they do all the harm above mentioned. Therefore as a part removal of hypertrophied tonsils will do in almost all cases, a part removal of the adenoid tumors is better than none, and therefore I endorse the proceeding that is the easiest and best. I say endorse because it is not mine. It is as old as the discovery of this pathological condition itself, and the only employed proceeding by the discoverer, Dr. William Meyer, of Copenhagen, himself. Is this pathological condition a common or, at least, very frequent evil, what most physicians agree upon; then it must be our aim to find some simple means for it, that are employable for every physician, and not stare us blind on an outfit that will make most of us hesitate before employing it. I trust that I am not fighting a spook. I have lately read many articles upon this subject, and nowhere found anything about this proceeding, save a passing notice ; but an operation with rhinoscopic mirror, mouth-gag, palate-retractor, chloroform and assistants and so forth has been the only thing worth mentioning in detail and with a minuteness as if this were the only operation in existence. It is all very well to look for some means wherewith the least pain is caused ; we ought all of us to be so kind-hearted, but for the sake of avoiding a little short pain, we are not justified in throwing away a proceeding that will in the end give just as good results as a complicated one, that only, very few specialists can and will employ. It is a pity, fellow physicians, to witness the above mentioned crippled condition in many children, what mostly is irreparable in later years, when we stop to think that, taken in time, it might easily have been prevented, by our only putting the index finger behind the soft palate.

For the sake of illustration, I shall only mention two recent cases, that will show conclusively what might be obtained with the above recommended proceeding.

CASE I.—Alice K., aged twelve years, is very hard of hearing. Fœtid discharge from both ears since she was about five years old. The mother of the patient has been told by several physicians that the child would outgrow it, and consequently nothing has been done except an occasional cleaning of the ears.

As the poor hearing puts her very much back at school, the mother decided not to wait any longer for the outgrowing, but consulted one of our best and most reputable throat specialists. As he was no ear doctor he advised her to go and see such a one first and then come back to him. It was thus she called on me.

After a thorough cleaning of the ears, the drum-heads are found destroyed and the hearing reduced to contact for the watch. She breathes mostly through the mouth, and this tells better than any examination her trouble. The stupid expression has spoiled for good a handsome face, but save for a slight setback to the development of her chest, she looks and feels strong and healthy.

In three sittings the adenoid vegetations were scraped away with the finger-nail, or most of them. Nothing specially done to the ears but cleaning. The hearing increased to about twelve inches for the watch in both ears, and free breathing is enjoyed still, although a subsequent examination with the rhinoscope a couple of months after revealed some small tumor rests in the post-nasal space.

The other patient (CASE II) was a lady of twenty years of age with exactly the same symptoms, only more marked, especially as to the stupid expression of her face. Here was also fœtid discharge from both ears, only for which she consulted me. The adenoid vegetations, filling up almost the pharyngeal vault, were removed with Meyer's ring-knife in three sittings—also here almost, as there is still to be seen a bridge of adenoid tissue from side to side right below the posterior edge of the septum—and the ears only cleaned and plugged with borated cotton. The breathing through the nose is entirely free and the hearing in both ears considerably increased. This illustrates, gentlemen, also the unpardonable folly of separating in practice the ear treatment from that of the nose and throat.

*70 State Street.*

